# Cell wall N-glycan of *Candida albicans* ameliorates early hyper- and late hypo-immunoreactivity in sepsis

**DOI:** 10.1038/s42003-021-01870-3

**Published:** 2021-03-16

**Authors:** Masataka Kawakita, Taiki Oyama, Ikuma Shirai, Shuto Tanaka, Kotaro Akaki, Shinya Abe, Takuma Asahi, Guangwei Cui, Fumie Itoh, Masato Sasaki, Nobuyuki Shibata, Koichi Ikuta, Tomomitsu Hatakeyama, Kazuhiko Takahara

**Affiliations:** 1 Department of Animal Development and Physiology, Graduate School of Biostudies, Kyoto University, Kyoto, Japan; 2 Laboratory of Immune Regulation, Department of Virus Research, Institute for Frontier Life and Medical Sciences, Kyoto University, Kyoto, Japan; 3 Graduate School of Medicine, Kyoto University, Kyoto, Japan; 4 Division of Infection and Host Defense, Tohoku Medical and Pharmaceutical University, Sendai, Japan; 5 Biomolecular Chemistry Laboratory, Graduate School of Engineering, Nagasaki University, Nagasaki, Japan

**Keywords:** Sepsis, Immune evasion

## Abstract

Severe infection often causes a septic cytokine storm followed by immune exhaustion/paralysis. Not surprisingly, many pathogens are equipped with various anti-inflammatory mechanisms. Such mechanisms might be leveraged clinically to control septic cytokine storms. Here we show that N-glycan from pathogenic *C. albicans* ameliorates mouse sepsis through immunosuppressive cytokine IL-10. In a sepsis model using lipopolysaccharide (LPS), injection of the N-glycan upregulated serum IL-10, and suppressed pro-inflammatory IL-1β, TNF-α and IFN-γ. The N-glycan also improved the survival of mice challenged by LPS. Analyses of structurally defined N-glycans from several yeast strains revealed that the mannose core is key to the upregulation of IL-10. Knocking out the C-type lectin Dectin-2 abrogated the N-glycan-mediated IL-10 augmentation. Furthermore, *C. albicans* N-glycan ameliorated immune exhaustion/immune paralysis after acute inflammation. Our results suggest a strategy where the immunosuppressive mechanism of one pathogen can be applied to attenuate a severe inflammation/cytokine storm caused by another pathogen.

## Abstract (web summary)

Kawakita et al use a murine sepsis model to show that N-glycan from pathogenic *C. albicans* ameliorates sepsis - and thus improves survival - through upregulation of the cytokine IL-10. This study demonstrates a potential strategy in which the immunosuppressive mechanism of one pathogen can be applied to attenuate a severe inflammation caused by another pathogen.

## Introduction

Infection leading to sepsis continues to be a major health concern^1,2^. It is estimated that 30 million people worldwide are affected each year. In sepsis, microbes and viruses cause severe inflammation accompanied by an excess production of proinflammatory cytokines, commonly referred to as a cytokine storm, resulting in multi-organ failure^3^. In many cases, after the inflammation is resolved, immune paralysis is induced, leading to the majority of septic deaths^1,2^. Therefore, practical methods to control these septic responses are urgently required.

IL-10 is a potent immunosuppressive cytokine that plays pleiotropic roles in the management of the immune system^4,5^. Various types of immune cells produce IL-10, and, in turn, suppress immune system activity by downregulating the production of proinflammatory cytokines, including IL-1β, TNF-α, and IFN-γ, which, when overproduced, generate the cytokine storm. IL-10 suppresses acute systemic inflammation, as in sepsis^6^, as well as T cell exhaustion^7^ that leads to hypo-immunoreactivity and recurrent/further infections. Furthermore, IL-10 also ameliorates acute lung inflammation and injury associated with influenza infection^8^. These properties make IL-10 and the IL-10 biosynthetic pathway attractive candidates for treatments of various types of diseases, such as colitis, arthritis and pancreatitis^9^. In principle, control of IL-10 expression in vivo could be extended to the treatment of severe inflammation and inflammatory diseases. Disappointingly, however, several trials of injection of exogenous IL-10 showed low efficacy and various side effects^5^, partly due to site and timing of its actions. As an alternative, induction of IL-10 in vivo could possibly improve these problems.

In the 1970’s, an immunosuppressive factor was found in the blood of candidiasis patients^10^. The factor comprised mannoproteins released from the cell wall of *Candida albicans*^10,11^, and it suppressed candida-induced proliferation of lymphocytes and delayed-type hypersensitivity (DTH) of the patients^10^. These effects were eliminated from patients by treatments with anti-mycotic agents^10^. In the mannoproteins, the polysaccharide mannan is indispensable for the immunosuppressive activities^12^. Thus, it is conceivable that the *C. albicans* evasion of the host immune system principally involves the immunosuppressive mannoprotein. However, identification of the effective mannan structure conferring the immunosuppressive activities remained elusive, primarily due to technical difficulties, e.g., chemical synthesis of polysaccharides.

In this report, we focused on *C. albicans* mannoprotein, for which immunosuppressive activities have been documented in candidiasis patients. Mannoprotein-derived N-glycan, purified from clinically isolated *C. albicans* strain J-1012, was tested in a mouse model where sepsis was induced by LPS. In this model system, N-glycan administration ameliorated early and late septic responses. Furthermore, the accumulation of structural information of N-glycans from several *Candida*/yeast strains enabled us to identify mannan structures that are indispensable for the activities.

## Results

### Amelioration of LPS-induced septic responses by *C. albicans* N-glycan

**Fig. 1:**
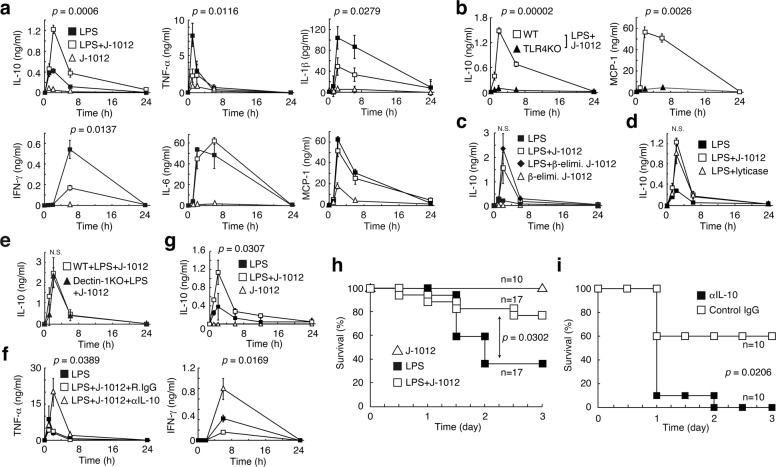
N-glycan of *C. albicans* up-regulates IL-10 production and improves mouse septic responses with LPS. **a**, **b** Serum cytokines after i.v. injection with a low dose of LPS (15 μg/20 g mouse weight) and J-1012 N-glycan (400 μg N-glycan/20 g mouse weight) in WT (**a**) or TLR4KO mice (**b**). **c**, **d** Serum IL-10 after i.v. injection with J-1012 N-glycan purified after β-elimination (**c**) or J-1012 N-glycan treated by lyticase in WT mice (**d**). **e** Effects of deficient of Dectin-1 on serum IL-10. **f** Effects of anti-IL-10 on serum cytokines. **g** Serum IL-10 after i.p. injection with a lethal dose (100 μg/20 g mouse weight) of LPS and J-1012 N-glycan. **h**, **i** Survival rates of mice after i.p. injection with J-1012 N-glycan and the high dose of LPS in the absence (**h**) or the presence (**i**) of mAbs, as indicated. These experiments were repeated twice, and compiled results are shown. **a**–**g** Data are expressed as the mean ± SD (*n* = 3) (**a**, **c**–**g**), (*n* = 6) (**b**) and are representative of at least two independent experiments. Statistical analyses were performed between LPS and LPS + J-1012 (**a**, **g**), LPS + J-1012 and LPS + J-1012 with β-elimination (**c**), LPS + J-1012 and LPS + J-1012 with lyticase (**d**), LPS + J-1012 + R.IgG and LPS + J-1012 + αIL-10 (**f**). *p* value at the peak point of cytokine production was determined by unpaired two-tailed Student’s *t*-test. N.S., not significant (*p* > 0.05). **h**, **i** *p* value was determined by the Wilcoxon test.

N-glycan was purified from mannoprotein of *C. albicans* strain J-1012 by Fehling’s method^13^ (hereinafter referred to as J-1012 N-glycan). Administration by i.v. injection of J-1012 N-glycan (400 μg N-glycan/20 g mouse weight) along with a low dose of LPS (15 μg/20 g mouse weight) resulted in up-regulation of IL-10 and down-regulation of proinflammatory IL-1β, TNF-α, and IFN-γ in serum, compared to administration of LPS alone (Fig. 1a). J-1012 N-glycan did not affect LPS-induced IL-6 and MCP-1production. The J-1012 N-glycan also augmented IL-10 production in vivo in response to a TLR2 ligand, Pam_3_CSK_4_, but showed up-regulation of TNF-α and IL-6 production (Supplementary Fig. 1). In response to LPS, J-1012 N-glycan alone induced small amounts of these cytokines. IL-10 production was not observed in TLR4 knockout (KO) mice, consistent with essential role of TLR4 in LPS recognition (Fig. 1b). This also indicates that the IL-10 production is driven by LPS rather than a possible contaminant in J-1012 N-glycan. β-elimination, which minimizes contamination of J-1012 N-glycan by O-glycan, did not affect the induction of IL-10 (Fig. 1c). Composition analysis of J-1012 N-glycan by gas chromatography detected contamination by small amounts (~3%) of glucose, possibly derived from β-glucan in the cell wall. However, lyticase treatment, which degrades β1,3-glucan, did not affect IL-10 induction (Fig. 1d). Furthermore, mice lacking the β-glucan receptor Dectin-1 produced comparable levels of IL-10 to those of WT mice in response to J-1012 N-glycan and LPS (Fig. 1e). To assess the correlation between IL-10 and the other cytokines, we co-injected neutralizing anti-IL-10 mAb along with J-1012 N-glycan and LPS. As a result, large amounts of TNF-α and IFN-γ were observed, consistent with the control of these cytokines by IL-10 (Fig. 1f). To test the efficacy of J-1012 N-glycan in a more challenging context, a lethal dose of LPS (100 μg/20 g mouse weight) was administered, and co-injection of mice with J-1012 N-glycan not only augmented serum IL-10 production (Fig. 1g), but improved survival (Fig. 1h). Neutralization of IL-10 by mAb negated the improvement (Fig. 1i). Taken together, these results indicate that J-1012 N-glycan augments production of IL-10 in vivo. In turn, elevated levels of IL-10 could suppress the cytokine storm, leading to a reduction of septic death.

### Involvement of α-mannan and mannose core of the N-glycan

**Fig. 2:**
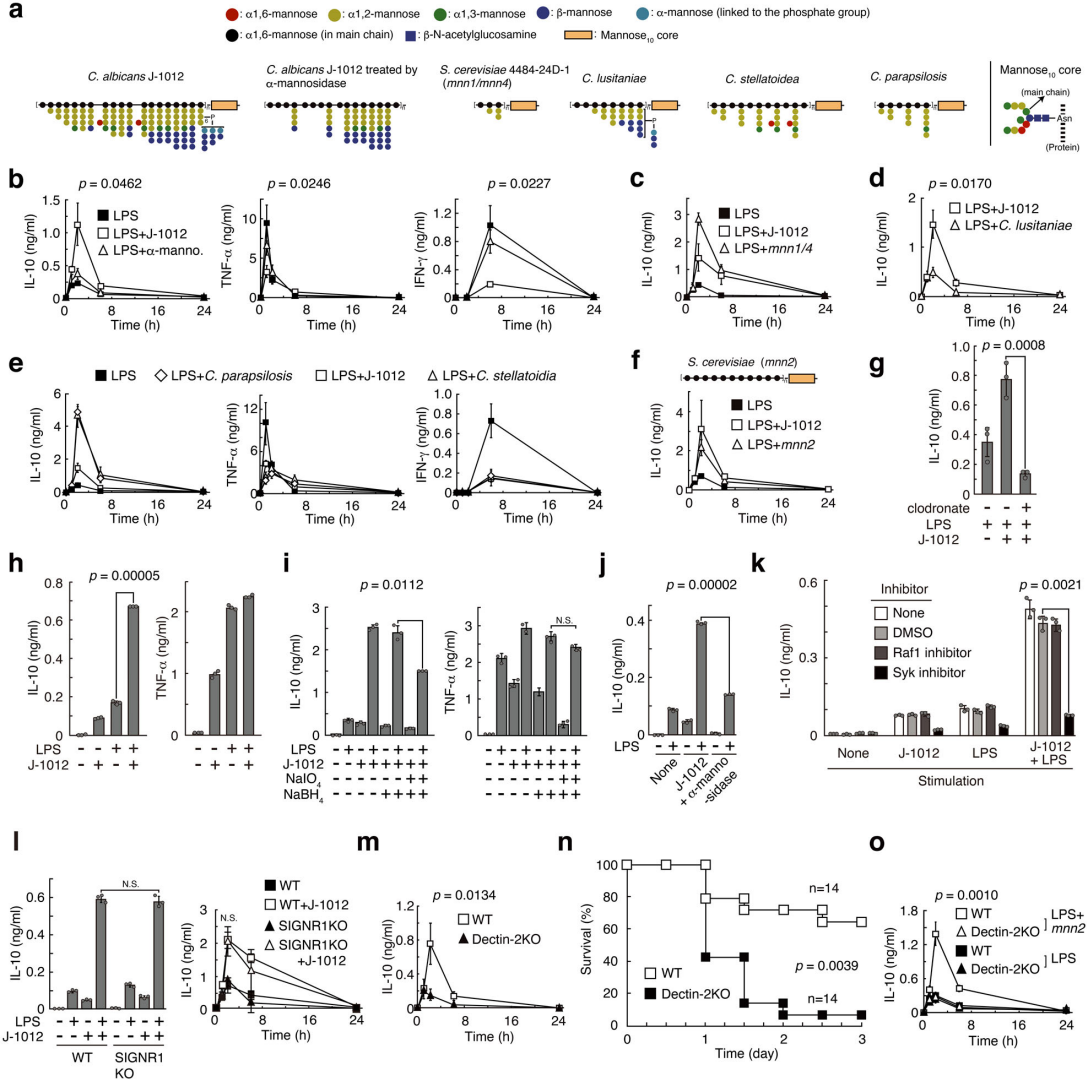
α-mannans of N-glycan of *C. albicans* are involved in augmentation of IL-10 through Dectin-2. **a** Schematic structures of N-glycans and mannose core (black circle; α1,6-mannose (in main chain), blue circle; β-mannose, green circle; α1,3-mannose, light blue circle; α-mannose (linked to the phosphate group), red circle; α1,6-mannose, yellow circle; α1,2-mannose, light brown rectangle; Mannose_10_ core). **b**–**f** Analyses of serum cytokines after i.v. injection with LPS and J-1012 N-glycan treated with α-mannosidase (**b**), *S. cerevisiae* (*mnn1/mnn4*) (**c**), *C. lusitaniae* (**d**), *C. parapsilosis* and *C. stellatoidea* (**e**), and *S. cerevisiae* (*mnn2*) (**f**) N-glycan as in Fig. 1a. **g** Serum IL-10 of 2 h after i.v. injection with LPS and J-1012 N-glycan in mice pre-treated by clodronate liposome. **h**–**k** Cytokine production by rpMϕ after stimulation by LPS for 24 h on plates coated with intact (**h**, **k**), NaIO_4_ treated (**i**) or α-mannosidase treated (**j**) J-1012 N-glycan. Effects of signaling inhibitors on IL-10 production by rpMϕ (**k**). **l** IL-10 production of SIGNR1KO rpMϕ in response to plate-coated J-1012 N-glycan (left panel) and serum IL-10 in SIGNR1KO mice as in Fig. 1a (right panels). **m** Serum IL-10 in Dectin-2KO mice upon stimulation with LPS in the presence of J-1012 N-glycan as in Fig. 1a. **n** Survival of WT and Dectin-2KO mice as in Fig. 1h. The experiment was repeated twice, and compiled results are shown. **o** Serum IL-10 in Dectin-2KO mice after injection with N-glycan from *S. cerevisiae* X2180-1A-5 (*mnn2*). **b**–**m**, **o** Data are expressed as the mean ± SD (*n* = 3) (**b**–**l**), (*n* = 4) (**m**) and (*n* = 4) (**o**), and are representative of at least two independent experiments. In (**b**), (**c**), (**l** right panel) and (**o**), statistical analyses were performed between LPS + J-1012 and LPS + J-1012 with α-mannosidase, LPS + J-1012 and LPS + *mnn1/4*, WT + J-1012 and SIGNR1KO + J-1012, and WT + *mnn2* + LPS and Dectin-2KO + *mnn2* + LPS, respectively. **b**, **d**, **l** (right panel), **m**, **o**, *p* value at the peak point of cytokine production was determined by unpaired two-tailed Student’s *t*-test. **g**–**l** *p* value was determined by unpaired two-tailed Student’s *t*-test. N.S., not significant (*p* > 0.05). **n** *p* value was determined by the Wilcoxon test.

We previously reported that J-1012 N-glycan contains various types of side chains (Fig. 2a), including α-mannan side chains (55.4% of total side chains), β-mannan-capped α-mannan side chains (41.9%) and phosphomannan side chains (2.7%)^13^. Specific structural patterns in the side chains may be involved in the augmentation of IL-10 production. To investigate this possibility, we first treated J-1012 N-glycan with α-mannosidase to remove α-mannan side chains, phosphomannan and mannose/Man_10_GlcNAc_2_ core (designated as Man_10_Core), but not β-mannan-capped α-mannan side chains (Fig. 2a). This treatment of J-1012 N-glycan reduced the IL-10 production and restored the TNF-α and IFN-γ induction by LPS in vivo (Fig. 2b), suggesting that there are structural features of J-1012 N-glycan that are sensitive to α-mannosidase. Moreover, *C. albicans* NIH B-792 N-glycan without β-mannan-capped α-mannan side chains showed the augmentation of IL-10 (Supplementary Fig. 2), implying the dispensability of the structure. We next tested N-glycan purified from *S. cerevisiae* 4484-24-D1 (*mnn1*/*mnn4*), which contains α1,2-mannan side chains, but not phosphomannan. This N-glycan induced larger amounts of IL-10 than J-1012 N-glycan did (Fig. 2c), underscoring the essential contribution of α-mannan side chains and/or Man_10_Core. This result also suggests that β-mannose-capped side chains in J-1012 N-glycan suppresses the IL-10 augmentation. We then tested N-glycan from *C. lusitaniae* (non-albicans strain), in which the side chains are predominantly β-mannan-capped α-mannan (77.5%) rather than α-mannan (14.9%) and phosphomannan (7.6%)^14^. Indeed, this N-glycan elicited significantly less IL-10 induction in vivo than J-1012 N-glycan (Fig. 2d). This result revealed the importance of α/β-mannan composition in the side chains. Following this line of reasoning, it is possible that N-glycan containing long α-mannan side chains without β-mannan-capped α-mannan side chains should efficiently induce IL-10. As expected, N-glycans isolated from *C. stellatoidea* (*Candida albicans* (Robin) Berkhout) and *C. parapsilosis* (non-albicans strain) (Fig. 2a) significantly induced large amounts of IL-10 in comparison with J-1012 N-glycan in the sepsis model (Fig. 2e). Both N-glycans showed TNF-α and IFN-γ levels equivalent to those induced by J-1012 N-glycan. On the other hand, *C. albicans* NIH B-792 N-glycan, whose structure is similar to that of *C. stellatoidea*, but with phosphomannan side chains consisting of β-mannan, showed the same extent of IL-10 augmentation as J-1012 N-glycan (Supplementary Fig. 2), suggesting that there are negative effects of β-mannan on IL-10 augmentation. These results indicated that the α-mannan structure in the side chains affects the augmentation of IL-10 production. However, N-glycan from *S. cerevisiae* X2180-1A-5 (*mnn2*)^15^, which consisted exclusively of an α1,6-mannan backbone and Man_10_Core still showed IL-10 augmentation in vivo (Fig. 2f), suggesting that there are effective contributions from 2 different linkages, as well as the α-mannan structure in the side chains.

To find more clues about the mannan structure required for IL-10 induction in vivo, we identified the host receptor for J-1012 N-glycan. We first sought to identify the cell type involved in the IL-10 production using clodronate-liposomes that eliminated phagocytic cells in vivo (Supplementary Fig. 3). This treatment significantly decreased IL-10 production in response to J-1012 N-glycan and LPS (Fig. 2g). In vivo treated resident peritoneal macrophages (rpMϕ) showed augmentation of IL-10 production by J-1012 (Supplementary Fig. 4). In vitro, purified rpMϕ grown on plates coated with J-1012 N-glycan produced large amounts of IL-10, but not TNF-α, after stimulation with LPS (Fig. 2h). The production of IL-10 was sensitive to treatment with NaIO_4_-NaBH_4_, which damages the saccharide structure (Fig. 2i), or α-mannosidase, which digests α-mannan in the J-1012 N-glycan (Fig. 2j). NaBH_4_ reducing aldehyde group generated by NaIO_4_ did not affect the IL-10 production alone. On phagocytic cells, there are several lectins that recognize yeast mannose/mannan^16^. To further narrow down the list of putative receptors for J-1012 N-glycan, the effects of various types of signaling inhibitors were tested, using the rpMϕ assay (Fig. 2k and Supplementary Fig. 5). Of these inhibitors, GW5074, a Raf-1 kinase inhibitor that suppresses IL-10 production via human DC-SIGN^17^, showed no effect on the IL-10 induction. In addition, SIGNR1, a homolog of human DC-SIGN, causing oral tolerance through IL-10 induction in intestine^18^, was dispensable for the augmentation of IL-10 production by J-1012 N-glycan in vitro and in vivo (Fig. 2l). On the other hand, piceatannol, a Syk kinase inhibitor, suppressed the IL-10 induction effectively (Fig. 2k). Of the lectins using Syk signaling, Dectin-2 is known to recognize α-mannan of *C. albicans*^19^. We therefore checked Dectin-2KO mice, and observed markedly impaired IL-10 induction by J-1012 N-glycan in vivo (Fig. 2m). Consistent with this observation, Dectin-2KO mice were significantly more susceptible to a lethal dose of LPS with J-1012 N-glycan (Fig. 2n). Furthermore, IL-10 augmentation by N-glycan from *S. cerevisiae* X2180-1A-5 (*mnn2*) also depended on Dectin-2 (Fig. 2o). Taking into consideration the fact that Dectin-2 does not recognize α-1,6 mannan^20^ in the backbone, these results suggest that Dectin-2 recognizes the mannose core of J-1012 N-glycan, leading to augmentation of IL-10 production in vivo.

### Cytokine induction by synthetic mannose core though Dectin-2

**Fig. 3:**
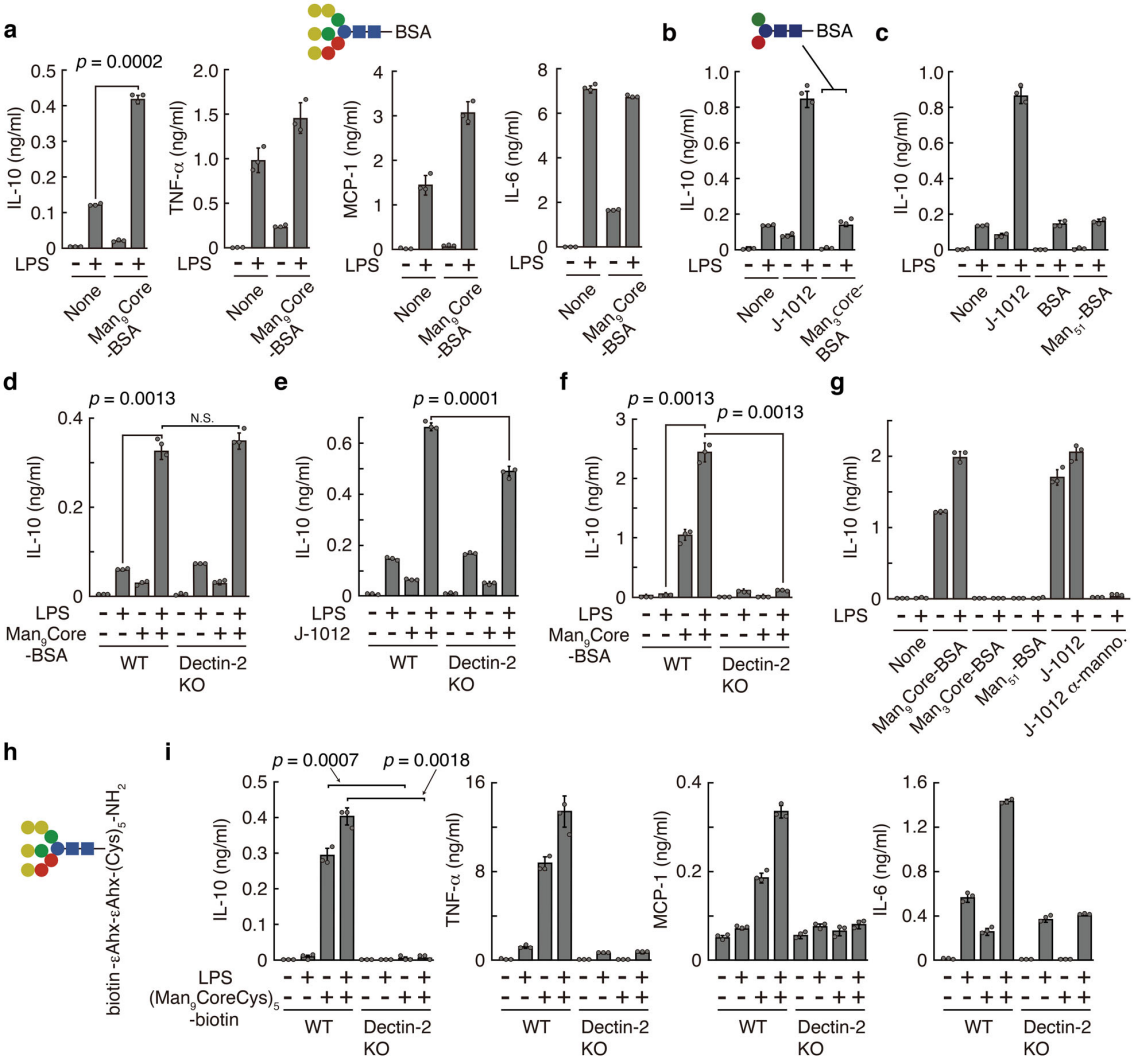
Augmentation of IL-10 production by mannose core in vitro. **a**–**e** Cytokine production by rpMϕ from WT(**a**–**c**) and Dectin-2KO (**d**, **e**) mice after stimulation with Man_9_Core-BSA, Man_3_Core-BSA, Man_51_-BSA (bound more than 51 mannose monomers), and J-1012 N-glycan, immobilized on plates as in Fig. 2h. Schematic structures of Man_9_Core-BSA and Man_3_Core-BSA are depicted. **f** IL-10 production by BMDCs from WT and Dectin-2KO mice in response to Man_9_Core-BSA. **g** IL-10 production by BMDCs from WT mice upon stimulation with Man_9_Core-BSA, Man_3_Core-BSA, Man_51_-BSA, J-1012 N-glycan, and J-1012 N-glycan treated with α-mannosidase. **h** Schematic structure of (Man_9_CoreCys)_5_-biotin. εAHX, ε-amino-n-hexanoic acid. **i** Cytokine production by BMDCs from WT and Dectin-2KO mice upon stimulation with (Man_9_CoreCys)_5_-biotin bound to an avidin-coated plate. These experiments were repeated at least twice, and representative results are shown. Data are expressed as the mean ± SD. *p* value was determined by unpaired two-tailed Student’s *t-*test. N.S., not significant (*p* > 0.05).

We next synthesized a Man_9_Core conjugated with BSA (Man_9_Core-BSA) and tested its ability to induce IL-10 in the rpMϕ system. Man_9_Core-BSA coated plates induced production of IL-10 and the other cytokines (Fig. 3a), while control conjugated Man_3_Core-BSA and mannose-BSA (Man_51_-BSA), which is known to induce oral tolerance through SIGNR1^18^, did not (Fig. 3b, c). Unexpectedly, however, the response of rpMϕ to Man_9_Core-BSA was independent of Dectin-2 (Fig. 3d). Moreover, the response of rpMϕ to J-1012 N-glycan depended on Dectin-2, but only to some extent (Fig. 3e). These results suggest that other receptors using Syk signaling, e.g., Mincle (Supplementary Fig. 6) and Dectin-3^16^, whose expression levels are higher in Mϕ than DCs, are involved in Man_9_Core recognition in vitro. On the other hand, the response of bone marrow-derived DCs (BMDCs) to Man_9_Core-BSA depended on Dectin-2 (Fig. 3f) and on CARD9, a member of the signalosome for Syk^21^ (Supplementary Fig. 7). BMDCs did not respond to Man_3_Core-BSA and Man_51_-BSA (Fig. 3g), similar to the lack of response in rpMϕ. The IL-10 production in response to Man_9_Core-BSA was down-regulated by J-1012 N-glycan in the medium, consistent with the notion that there is steric hindrance between the mannose core and the N-glycan (Supplementary Fig. 8) Furthermore, biotinylated penta-Man_9_Core ((Man_9_CoreCys)_5_-biotin), high-density Man_9_Core with a minimum carrier (Fig. 3h), stimulated IL-10 production by BMDCs in a Dectin-2-dependent manner (Fig. 3i). In addition, the (Man_9_CoreCys)_5_-biotin also induced TNF-α, MCP-1 and IL-6 production from BMDCs in a Dectin-2-dependent manner. These results with synthetic glycans further implicate Dectin-2 in the recognition of the mannose core in the N-glycan. Collectively, our results suggest that the mannose core of *C. albicans* N-glycan is recognized by Dectin-2, leading to the IL-10 augmentation and subsequent suppression of the early onset of septic responses in vivo.

### Amelioration of hypo-immunoreactivity by *C. albicans* N-glycan

**Fig. 4:**
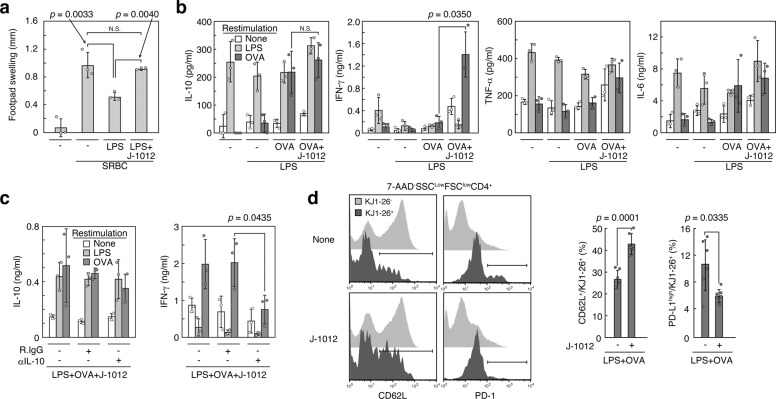
Amelioration of hypo-immunoreactivity by J-1012 N-glycan. **a** Recovery of delayed-type hypersensitivity in mouse footpad by J-1012 N-glycan. **b**, **c** Analyses of cytokine production by splenocytes from mice after 2 weeks of sepsis induction with i.v injection of OVA, J-1012 N-glycan and LPS without (**b**) or with anti-IL-10 mAb (**c**). The splenocytes were restimulated as described for 4 days. **d** Expression of CD62L and PD-1 of transferred DO11.10 T cells (CD4^+^KJ1-26^+^) (Supplementary Fig. 10) from mice after 2 weeks of sepsis induction with OVA, LPS and J-1012 N-glycan. The experiment was repeated twice, and compiled results are shown (right panels) (*n* = 6). **a**–**c** Data are expressed as the mean ± SD and are representative of at least two independent experiments (*n* = 3). *p* value was determined by unpaired two-tailed Student’s *t*-test. N.S., not significant (*p* > 0.05). R.IgG, control rat IgG. SRBC sheep red blood cells.

Hypo-immunoreactivity/immune paralysis is often observed after several days or weeks of sepsis, leading to recurrent and opportunistic infections followed by septic death in the majority of cases^1^, and to an increased risk of cancer development in humans^22^. The secondary infection is also the case with severe virus infection^23^. At present, there is no practical treatment for the hypo-immunoreactivity. It is also possible that the IL-10 augmentation by J-1012 N-glycan affects the hypo-immunoreactivity after the resolution of acute inflammation. We therefore investigated the effects of the J-1012 N-glycan on the hypo-immunoreactivity response. First, DTH response was evaluated. DTH is caused by antigen-specific effector T cells pre-sensitized. After challenge with the same antigen, the T cells induce productions of cytokines including IFN-γ and trigger type IV hypersensitivity, e.g., skin swelling. We first observed that i.v. LPS injection immediately after sensitization with sheep red blood cells (SRBC) suppressed the DTH response in footpad after 1 week, indicating hypo-immunoreactivity after sepsis induction (Fig. 4a). This weak response was reversed by co-injection of LPS with the J-1012 N-glycan concomitant with the SRBC sensitization, suggesting improvement of the hypo-immunoreactivity, instead of aggravation. We next observed the effects of the J-1012 N-glycan on antigen-specific IFN-γ production several weeks after sepsis induction, because pre-clinical adjunctive immunotherapy involving IFN-γ seems to improve the hypo-immunoreactivity^24^. After 2 weeks of sepsis induction in the presence of ovalbumin (OVA) as an antigen, cytokine production of splenocytes was analyzed in vitro, showing that the J-1012 N-glycan enhanced antigen-specific IFN-γ production, which possibly supported a Th1 reaction in DTH^25^; notably, however, IL-10, TNF-α, and IL-6 production were not enhanced (Fig. 4b). The elevated IFN-γ levels were also observed 1 month after sepsis induction (Supplementary Fig. 9). The IFN-γ enhancement after 2 weeks was prevented by co-injection of neutralizing mAb to IL-10 along with J-1012 N-glycan and LPS (Fig. 4c), suggesting that the initial augmentation of IL-10 by J-1012 N-glycan was involved in the IFN-γ production after alleviating the inflammation. We next checked the effects of J-1012 N-glycan on T cell phenotypes after septic inflammation. After transfer of transgenic DO11.10 T cells, sepsis was induced with OVA and J-1012 N-glycan. After 2 weeks, DO11.10 T cells were analyzed. In spleen, the effects of J-1012 N-glycan on the number of DO11.10 T cells were minimal (Supplementary Fig. 10a, b). However, the expression levels of various types of surface proteins relative to T cell functions were altered; we observed an increased CD62L^+^ and a reduced PD-1^high^ population in comparison with LPS alone (Fig. 4d and Supplementary Fig. 10c). CD62L is involved in homing of T cells into lymph nodes to induce immune responses, and PD-1, an immune checkpoint receptor, negatively regulates T cell activation. PD-1 is observed on exhausted T cells^26^ and its expression is upregulated in septic patients^27–29^. On the other hand, expression of Dectin-2 on T cells in lymph node and spleen was undetectable before or after stimulation with i.v. injection of LPS (Supplementary Fig. 11), suggesting that there is no direct influence of the N-glycan on T cells. Moreover, 2 weeks after sepsis induction, MHC class II expression and the M1/M2 balance of Mϕ in spleen seemed identical in the presence or absence of J-1012 N-glycan (Supplementary Fig. 12). Taken together, it is possible that J-1012 N-glycan improves the late hypo-immunoreactivity by protecting T cells from excessive activation through the early augmentation of IL-10 production.

## Discussion

Our approach, based on a putative immune suppressing mechanism of *C. albicans*, points to its potential to control IL-10 production leading to amelioration of both the early hyper- and late hypo-immunoreactivity in sepsis. Clinically, sepsis treatment has to manage both phases. Nowadays, extensive care enables us to suppress the early hyper-inflammation. However, the suppression doesn’t improve the late hypo-immunoreactivity. As a result, the hypo-immunoreactivity has become the major cause of septic death. To avoid the hypo-immunoreactivity, some cytokines, e.g., IFN-γ, are used to maintain immunocompetence. However, it is difficult to use the cytokines because there are few markers to monitor the septic phase, in hyper or hypo-immunoreactivity, of the patient. From this point of view, the bidirectional properties of the N-glycan may lead to methods for biphasic sepsis treatment. On the other hand, the current understanding of sepsis is based on a balance between a systemic inflammatory response syndrome (SIRS) and a compensatory anti-inflammatory response syndrome (CARS). It is believed that CARS caused by IL-10 and the other immunosuppressive cytokines induces immune paralysis. However, at least in our system, large amounts of IL-10 in early sepsis did not induce immune paralysis. Instead, IL-10 augmented by N-glycan maintained immunoreactivity, suggesting that the origin and local environment, rather than the total amount of IL-10 production, affects subsequent immune competence after alleviation of the acute inflammation. From this point of view, the α-mannans in the N-glycan are expected to directly act on DCs, a center of the immune system, through Dectin-2. This may be why the N-glycan can ameliorate the septic cytokine storm and maintain T cell reactivity in vivo. Our future studies will move forward on artificial neo-glycan conjugates including mannose core and α-mannan side chains, and on testing their effects on acute inflammation caused by microbial and viral infection.

We observed in vivo IL-10 augmentation by N-glycans from several non-albicans candida strains and *S. cerevisiae* strains as well as three *C. albicans strains*, J-1012, NIH B-792, and *C. stellatoidea*. Therefore, the IL-10 augmentation depends on the structure of N-glycans rather than yeast strains. In the N-glycans, the composition of the side chains may affect their IL-10 augmentation capacity. Our results suggest that α-mannan and β-mannose-capped α-mannan side chains positively affect and inhibit IL-10 augmentation by the mannan core, respectively. These results are informative for the design of artificial neo-glycan conjugates.

The glycans likely will act in humans because the mannoprotein was originally found to be immunosuppressive in candidiasis patients. Dectin-2 may be a keystone to control both the hyper-and hypo-immunoreactivity at once. Dectin-2 is also a pharmaceutical target to manipulate IL-10 production in vivo, and to treat chronic and refractory candidiasis. At present, we do not know how intracellular Dectin-2-Syk signaling enhances cytokine production by LPS. Co-operation with the other receptors is also possible. Furthermore, stimulation of Dectin-2 by mannose core causes not only IL-10 production, but also the other cytokines’ productions in vitro. It will be important to identify the major IL-10- and IFN-γ-producing cells, and to establish how Dectin-2 is involved in the augmentation of IL-10 production and suppression of the other cytokines in vivo.

## Methods

### Ethics statement

All animal experiments were approved by Animal Research Committee, Graduate School of Biostudies, Kyoto University (protocol number Lif-K20008).

### Mice

Balb/c and C57BL/6 mice were obtained from Japan SLC (Hamamatsu, Japan). SIGNR1KO (C57BL/6 background) mice obtained from the Consortium for Functional Glycomics were back-crossed more than 8 generations to Balb/c mice. Dectin-1KO and Dectin-2KO mice were obtained from Dr. Yoichiro Iwakura (Tokyo University of Science, Noda, Japan). TLR4KO mice (Balb/c background) were kind gifts from Dr. Akira Matsumoto (Ehime University, Ehime, Japan). Mice expressing DO11.10 TCR were obtained from the Institute for Frontier Medical Sciences (Dr. Shimon Sakaguchi, Kyoto University). Male and female mice were maintained under specific pathogen-free conditions and used at 8–12 weeks of age. All experiments were conducted according to our institutional guidelines.

### Antibodies and reagents

Biotin-labeled anti-B7-H1 (MIH5), B7-DC (TY25), BTLA (6F7), CD8 (53-6.7), CD44 (1M7), CD95L (MFL3), CD152 (UC10-4B9), GITR (DTA-1), PD-1 (J43), Armenian hamster IgG and rat IgG_2a_κ (eBR2a) were obtained from eBioscience (San Diego, CA). Biotin-labeled anti-CD4 (GK1.5), CD11c (N418), CD16/32 (2.4G2), CD19 (1D3), CD28 (37.51), CD49b (DX5), CD80 (16-10A1), CD86 (GL1), CD122 (5H4), CD127 (B12-1), B220 (RA3-6B2), Gr-1 (RB6-8C5), I-A^d^ (AMS-32.1), IL-10 (JES5-16E3), LAG3 (C9B7W), Mac1 (M1/70), γδTCR (GL3) and rat IgG_1_κ (R3-34) were obtained from BD Pharmingen (La Jolla, CA). For flowcytometry, these biotin-labeled antibodies were used in combination with avidin-DyLight 649, PE-Cy7-labeled streptavidin (BioLegend, San Diego, CA) and Alexa647-labeled avidin (Jackson Laboratory, Bar Harbor, ME). FITC-labeled anti-CD4, FITC-labeled control ratIgG_2_aκ (R35-95), PE-labeled anti-CD11b (M1/70), PE-labeled anti-CD86 (GL1), PE-labeled anti-DO11.10 TCR (KJ1-26), APC-labeled anti-CD4 (53-6.7) and PE-labeled hamster IgG_1_λ_1_ (G235-2356) were obtained from BD Pharmingen. FITC-labeled anti-Dectin-2 (KVa7-6E7) was obtained from Miltenyi Biotec (Bergisch Gladbach, Germany). Alexa488-labeled anti-CD206 (C068C2), FITC-labeled anti-CD11c (N418), APC-labeled F4/80 (BM8), APC-labeled rat IgG_2_aκ (RTK2758) were obtained from BioLegend. FITC- and APC-labeled F4/80 (BM8), APC-labeled anti-CD11c (N418), APC-labeled anti MHC II (M5/114.15.2), PE-labeled anti-CD3e (145-2C11), PE-labeled anti-CD80 (16-10A1), PE-labeled rat IgG_2a_κ (eBR2a) were obtained from eBioscience. Anti-Mincle (1B6) was obtained from MBL (Nagoya, Japan). Anti-IL-10 mAb (JES5-16E3) and control rat IgG (2A3) for neutralization experiments were obtained from Bio X Cell (West Lebanon, NH) and Jackson ImmunoResearch Laboratories (West Grove, PA), respectively. Ultra-pure *E. coli* LPS (0111:B4) (1 × 10^6^ EU/mg) was obtained from Invivogen (San Diego, CA) and used in all experiments. Pam_3_CSK_4_ was obtained from Calbiochem (San Diego, CA).

### Preparation of glycan from yeast

Cell wall mannoprotein was extracted from the acetone-dried cells using deionized water at 120°C for 2 h. The extract was dialyzed against water, concentrated and Fehling’s solution was added under stirring. After 5 min, insoluble copper chelate of the mannan was collected by centrifugation. The copper ion in the complex was then removed by using cation-exchange resin (Amberlite IR120, H^+^ form) (Sigma-Aldrich, St. Louis, MO). Resultant glycan (mannan) solution was dialyzed and lyophilized^30^. The glycans used in this study were purified from strains of *C. albicans* J-1012 (serotype A, *Candida albicans* (Robin) Berkhout, NBRC1060)^13^, *C. albicans* NIH B-792 (serotype B, *Candida albicans* (Robin) Berkhout, NBRC10108)^31^, *C. stellatoidea* (*Candida albicans* (Robin) Berkhout, NBRC 1397)^30^, *C. parapsilosis* (NBRC 1396)^31^, *C. lusitaniae* (NBRC1019)^14^, *S. cerevisiae* (*mnn1/4*)^32^ and *S. cerevisiae* X2180-1A-5 (*mnn2*)^15^. Glucose contamination of the glycan from *C. albicans* J-1012 was 2.8 ± 0.58% (*n* = 3) by composition analysis using gas chromatography. To remove O-glycan, the mannoprotein was treated with alkali (β-elimination)^33^. Briefly, the mannoprotein in 100 mM NaOH was kept at 25°C for 18 h. After neutralization and desalting, glycan was further purified by Fehling’s method.

The β-1,3-glucanase treatment of J-1012 N-glycan was carried out using a cell lytic enzyme, lyticase from *Arthrobacter luteus* (Zymolyase-100T, Nacalai Tesque, Kyoto, Japan) (EC3.2.1.6). J-1012 N-glycan was dissolved in 50 mM sodium phosphate buffer (pH 7.5) and incubated at 28°C for 3 h with 100 units/ml of the enzyme.

The α-mannosidase treatment of J-1012 N-glycan was carried out in 50 mM sodium acetate buffer (pH 4.6) containing 20 units of α-mannosidase (EC3.2.1.24) (Sigma-Aldrich, St. Louis, MO) at 37°C for 48 h.

### Periodate treatment of N-glycan

N-glycan coated on a plastic culture plate was treated with 50 mM of NaIO_4_ (Sigma-Aldrich) for 24 h at 4°C to cleave C2-C3 bonds in glucopyranose ring, and subsequently with 50 mM of NaBH_4_ (Sigma-Aldrich) for 2 h at room temperature to reduce the end generated. After washing the plate, purified rpMϕ were seeded and stimulated as described above.

### Synthetic mannose/mannan-BSA and -biotin

Man_9_Core-conjugated with bovine serum albumin (BSA) (17-22 residues in a BSA molecule) (Fig. 3a) and Man_3_GlcNAc_2_-conjugated with BSA (15 residues in a BSA molecule) (Fig. 3b) were synthesized by GlyTech, Inc. (Kyoto, Japan). Mannose-conjugated BSA (Man_51_-BSA) bound with more than 51 mannose monomers was kindly provided by Dr. Shau-Ku Huang (Taipei Medical University, Taipei, Taiwan). (Man_9_CoreCys)_5_-conjugated with biotin (Fig. 3h) was synthesized by GlyTech, Inc.

### Induction of endotoxin shock and cytokine production from splenocytes

For low-dose endotoxin shock, mice were injected i.v. with HANKS’ balanced salt solution (HBSS) (200 μl/20 g mouse weight) containing 75 μg/ml of LPS or 75 μg/ml of Pam_3_CSK_4_ with or without 2 mg/ml of N-glycan from *C. albicans* J-1012 and other strains. Serum cytokines were analyzed as described below. In some cases, mice were simultaneously injected i.v. with OVA (200 μg/20 g mouse weight), anti-IL-10 mAb (50 μg/20 g mouse weight) and control rat IgG. For analyses of in vitro cytokine production, after 2 weeks or as indicated, splenocytes (7.5 × 10^5^) were stimulated with LPS (100 ng/ml) or OVA (100 μg/ml) for 4 days in IMDM containing 10% FCS, antibiotics and 50 μM of β-mercaptoethanol. Cytokine production was analyzed as described below. For lethal endotoxin shock, mice were injected i.p. with HBSS (200 μl/20 g mouse weight) containing 500 μg/ml of LPS with or without 2 mg/ml of J-1012 N-glycan.

### Analyses of cytokine production

Cytokines in serum/culture supernatant were assessed by a Cytometric Bead Array mouse inflammatory kit and Mouse IL-1β Flex Set (BD Biosciences, Franklin Lakes, NJ) in accordance with the manufacturers’ protocols using a FACSCalibur system and an Accuri C6 Plus (BD Biosciences).

### Treatment of mice with clodronate liposomes

To deplete phagocytic cells in vivo, mice were treated i.v. and i.p. with anionic clodronate liposomes (FormuMax Scientific, CA) (60 μl/20 g mouse weight, respectively. Total 120 μl). After 24 h, elimination of phagocytic cells in spleen and peritoneal cavity was analyzed by flowcytometry, and sepsis was induced with the aforementioned low dose of LPS.

### Purification of rpMϕ, and stimulation with J-1012 N-glycan and synthetic mannans

rpMϕ were negatively enriched by depleting CD3ε, B220, CD19, Gr-1, and CD49b-expressing cells using biotinylated mAbs with avidin-IMAg (BD Pharmingen) from peritoneal cells^34^. Plastic plates were coated with 50 μg/ml of N-glycans and mannose/mannan-BSA for 12 h. Purified rpMϕ (5 × 10^4^ cells) were cultured in RPMI1640 containing 10% FCS, and 50 μM β-mercaptoethanol, and stimulated with LPS (100 ng/ml) for 24 h. In some cases, rpMϕ were pretreated with signaling inhibitors (Supplementary Table 1) for 1 h before stimulation with LPS.

### Preparation and stimulation of BMDCs

Bone marrow-derived DCs (BMDCs) were prepared from Balb/c bone marrow cells unless otherwise indicated^35^. Briefly, bone marrow was collected from femurs of adult mice and cultured in RPMI containing 10% FCS and 250 U/ml GM-CSF (gifted from Kirin Brewery). After 3 day in culture, equal volume of new media was added. On 6 day and 8 day, half of culture medium was replaced with new medium. Cells were harvested on 12 day. For CARD9KO BMDCs, bone marrow cells (C57BL/6 background) were kind gifts from Dr. Hiromitsu Hara (Kagoshima University, Kagoshima, Japan). BMDCs were purified using anti-CD11c-magnetic beads (Miltenyi Biotech, Bergisch Gladbach, Germany). For cytokine production analyses, BMDCs (1 × 10^5^ cells) were stimulated with LPS (100 ng/ml) for 24 h, in some cases, in the presence of J-1012 N-glycan (100 μg/ml) in medium. Biotinylated (Man_9_CoreCys)_5_ was immobilized on an avidin-coated culture plate (Thermo Scientific, MA) for 12 h at 10 μg/ml.

### Analyses of delayed-type hypersensitivity (DTH)

For DTH analyses, mice were immunized by injection of 2 × 10^6^ SRBC in 25 μl of HBSS into the left hind footpad and subsequently treated i.v. with HBSS (200 μl/20 g mouse weight) containing 75 μg/ml of LPS with or without 2 mg/ml of J-1012 N-glycan. After 7 days, the mice were given 1 × 10^8^ SRBC in 25 μl of HBSS into the right hind footpad. The footpad swelling was measured with a dial gauge caliper (Mitutoyo Corporation, Kanagawa, Japan) before and 24 h after antigen challenge.

### Transfer experiment of DO11.10 CD4 T cells

CD4 T cells were negatively enriched by depleting B220, CD8, CD11c, CD16/32, CD19, Gr-1, CD49b, I-A^d^, Mac1, γδTCR-expressing cells using biotinylated mAbs from lymph node cells of DO11.10 TCR transgenic mice, and transferred i.v. into Balb/c mice (2.5 × 10^6^ cells). After 24 h, the mice were subjected to endotoxin shock with OVA as described. After 2 weeks, the transferred cells were identified as 7-AAD^−^CD4^+^KJ1-26^+^.

### Analyses of Dectin-2 expression on Mϕ, DCs and T cells in lymph nodes and spleen

Single cells from superficial lymph nodes and spleen of WT and Dectin-2 mice were treated with anti-CD16/32 followed by flowcytometry. Mϕ, DCs and T cells were identified as CD11b^+^F4/80^+^7-AAD^−^, CD11c^+^7-AAD^−^ and CD3ε^+^CD4^+^/CD8^+^7-AAD^−^, respectively. In some cases, WT mice were pre-treated with i.v. injection of LPS (15 μg/20 g mouse weight) for 2 h.

### Intracellular staining

For IL-10 staining of rpMϕ, mice were i.p. treated with LPS and J-1012 N-glycan as in Fig. 1g. After 1 h, peritoneal cells were cultured in the presence of GolgiPlug for 5 h followed by intracellular staining with anti-IL-10 using Cytofix/Cytoperm Fixation/Permeabilization Kit (BD Pharmingen). For identification of M1 and M2 type Mϕ in spleen, splenocytes were first stained with biotin-labeled anti-CD86 and PE-Cy7-labeled streptavidin, and subsequently stained with Alexa488-labeled anti-CD206 intracellularly.

### Statistical analysis

Data are expressed as the means ± SD of triplicate assays. Statistical significance was determined by the two-tailed Student’s *t*-test. Differences in the survival of each group were determined by the Wilcoxon test. All experiments were performed twice or more times and representative results are shown. In some experiments, compiled results are shown.

### Reporting summary

Further information on research design is available in the Nature Research Reporting Summary linked to this article.

## Supplementary information

Supplementary Information

Description of Additional Supplementary Files

Supplementary Data 1

Supplementary Data 2

Supplementary Data 3

Supplementary Data 4

Supplementary Data 5

Reporting Summary

## Data Availability

All raw data is stored and securely backed-up and available upon request. All data underlying the graphs are available as Supplementary Data 1–5. Other data are available on reasonable request from the corresponding author.
