# Supplementary material for: Cell wall N-glycan of Candida albicans ameliorates early hyper- and late hypo-immunoreactivity in sepsis
Source: Commun Biol. 2021 Mar 16;4:342. doi: 10.1038/s42003-021-01870-3 (PMC7966402; doi:10.1038/s42003-021-01870-3)
Supplement: Supplementary file 1 — Supplementary Information [file 42003_2021_1870_MOESM1_ESM.pdf]

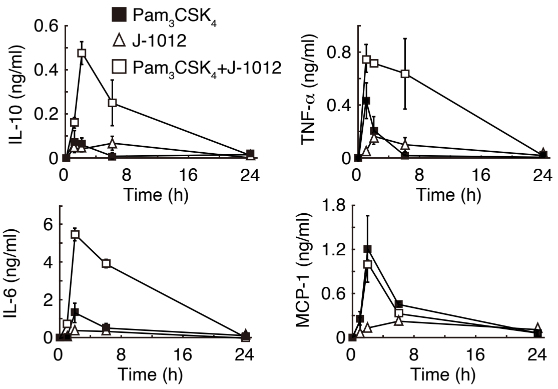

Supplementary Figure 1| Effects of J-1012 N-glycan on cytokine production in response to stimulation by Pam$_3$CSK$_4$ *in vivo*.

Serum cytokines were analyzed in the presence of Pam$_3$CSK$_4$ (20 μg/20 g body weight) as in Fig. 1a. Data are expressed as the mean ± SD (n=3) and are representative of two independent experiments.

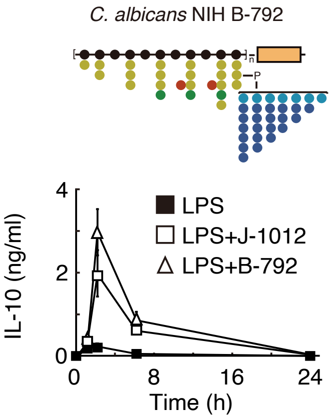

Supplementary **Figure 2| Schematic structure of N-glycan of *C. albicans* NIH B-792 and its up-regulation of IL-10 production *in vivo*.**

Serum cytokines after *i.v.* injection of N-glycan from NIH B-792 with LPS as in Fig. 1a. For the schematic structure, see Fig. 2a. Data are expressed as the mean ± SD (n=4) and are representative of two independent experiments.

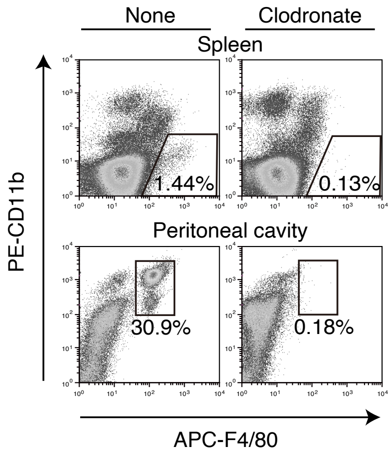

Supplementary Figure 3| Depletion of phagocytic cells by clodronate liposome.
After 24 h of *i.p.* and *i.v.* injection of clodronate liposomes, cells in the spleen and peritoneal cavity were analyzed by flow cytometry.

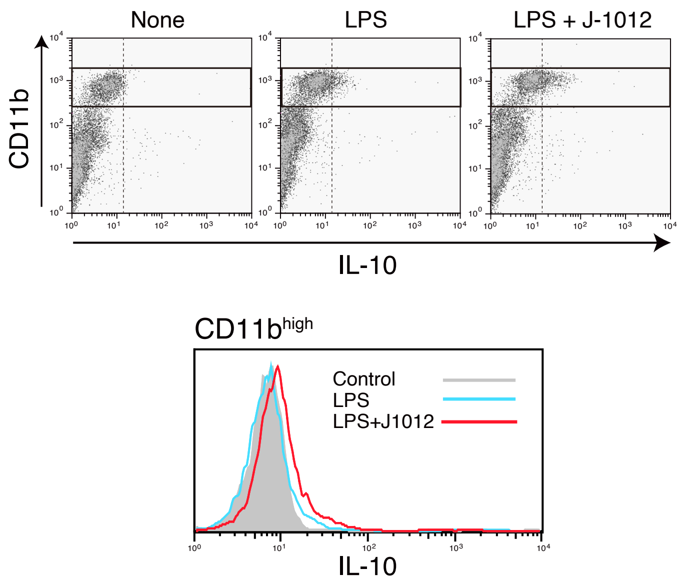

Supplementary Figure 4| Augmentation of IL-10 production in rpMϕ by J-1012 N-glycan.
Mice were *i.p.* treated with LPS and J-1012 N-glycan as in Fig. 1a. After 1 h, peritoneal cells were cultured in the presence of GolgiPlug for 5h followed by intracellular

staining with anti-IL-10. Experiments were repeated twice, and representative results are shown.

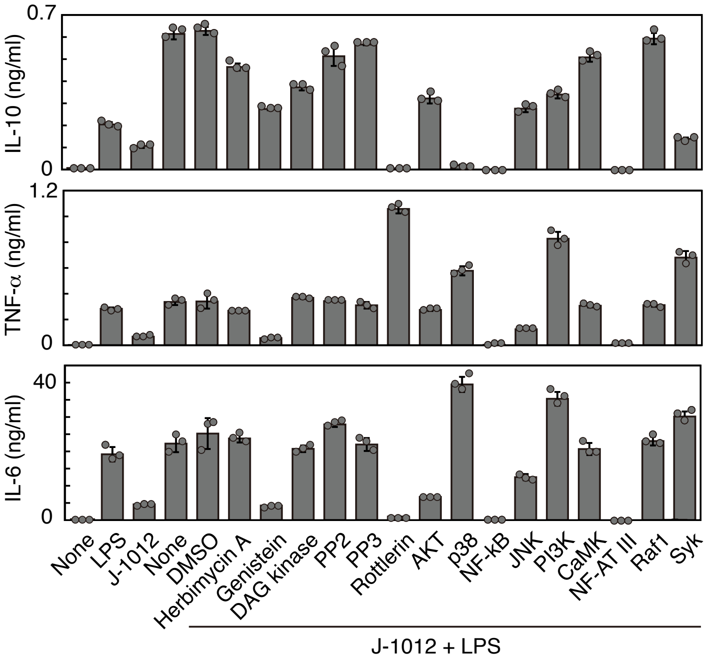

Supplementary Figure 5| Inhibition of rpMϕ IL-10 production by various types of signaling inhibitors *in vitro*.

Analyses of effects of signaling inhibitors (see Table S1) on IL-10 production by rpMϕ as in Fig. 2k. Data are expressed as the mean ± SD (n=3) and are representative of at least two independent experiments.

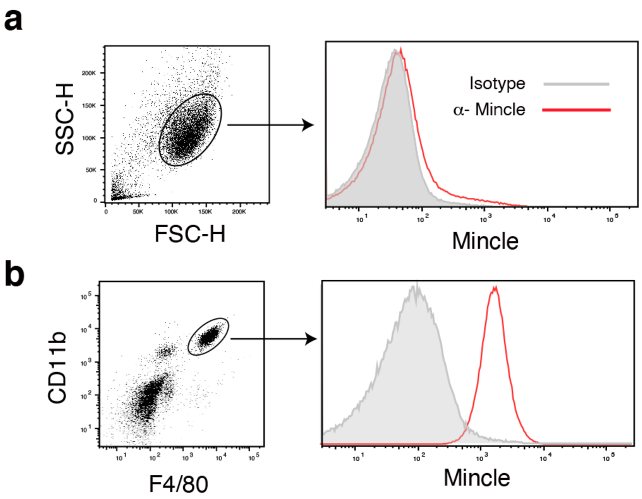

Supplementary Figure 6| Expression of Mincle in BMDCs and rpMϕ.

Mincle expression in BMDCs (**a**) and rpMϕ (**b**) was analyzed by flow cytometry. Experiments were repeated twice, and representative results are shown.

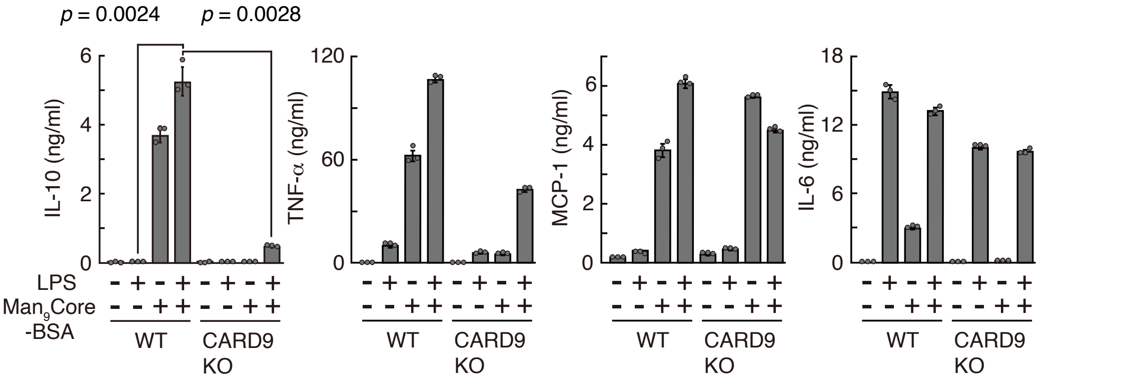

Supplementary Figure 7| Cytokine production of CARD9-deficient BMDCs in response to Man$_9$Core-BSA.

Cytokine production by BMDCs from WT and CARD9KO mice in response to Man$_9$Core-BSA was analyzed as in Fig. 3f. Data are expressed as the mean ± SD (n=3) and are representative of at least two independent experiments. *p* value was determined by unpaired two-tailed Student's *t*-test.

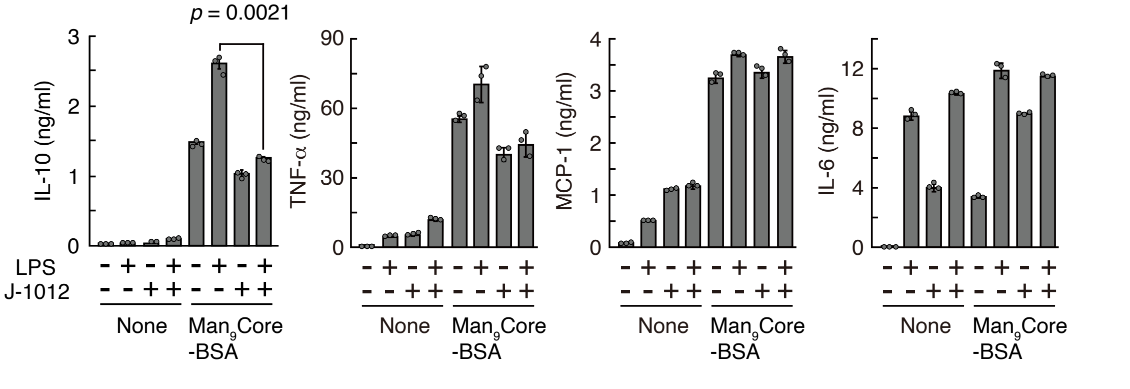

Supplementary Figure 8| Inhibition of cytokine production from BMDCs in response to Man$_9$Core-BSA by J-1012 N-glycan.

Cytokine production by BMDCs in response to immobilized Man$_9$Core-BSA was analyzed in the presence or absence of J-1012 N-glycan (100 μg/ml) in the medium as

in Fig. 3f. Data are expressed as the mean ± SD (n=3) and are representative of at least two independent experiments. *p* value was determined by unpaired two-tailed Student's *t*-test.

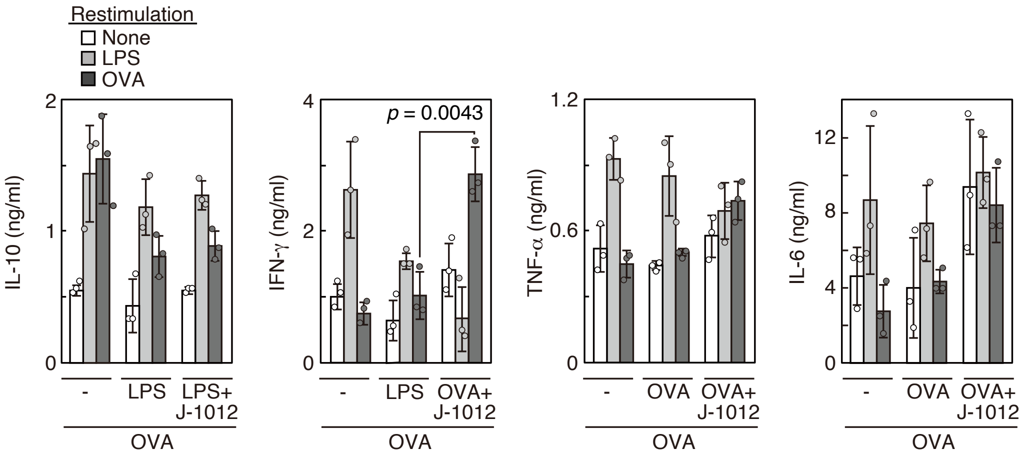

Supplementary Figure 9| Enhancement of IFN-γ production by J-1012 N-glycan. Analyses of cytokine production by splenocytes from mice pretreated 1 month before as in Fig. 4b. Data are expressed as the mean ± SD (n=3) and are representative of at least two independent experiments. N.S., not significant. *p* value was determined by unpaired two-tailed Student's *t*-test.

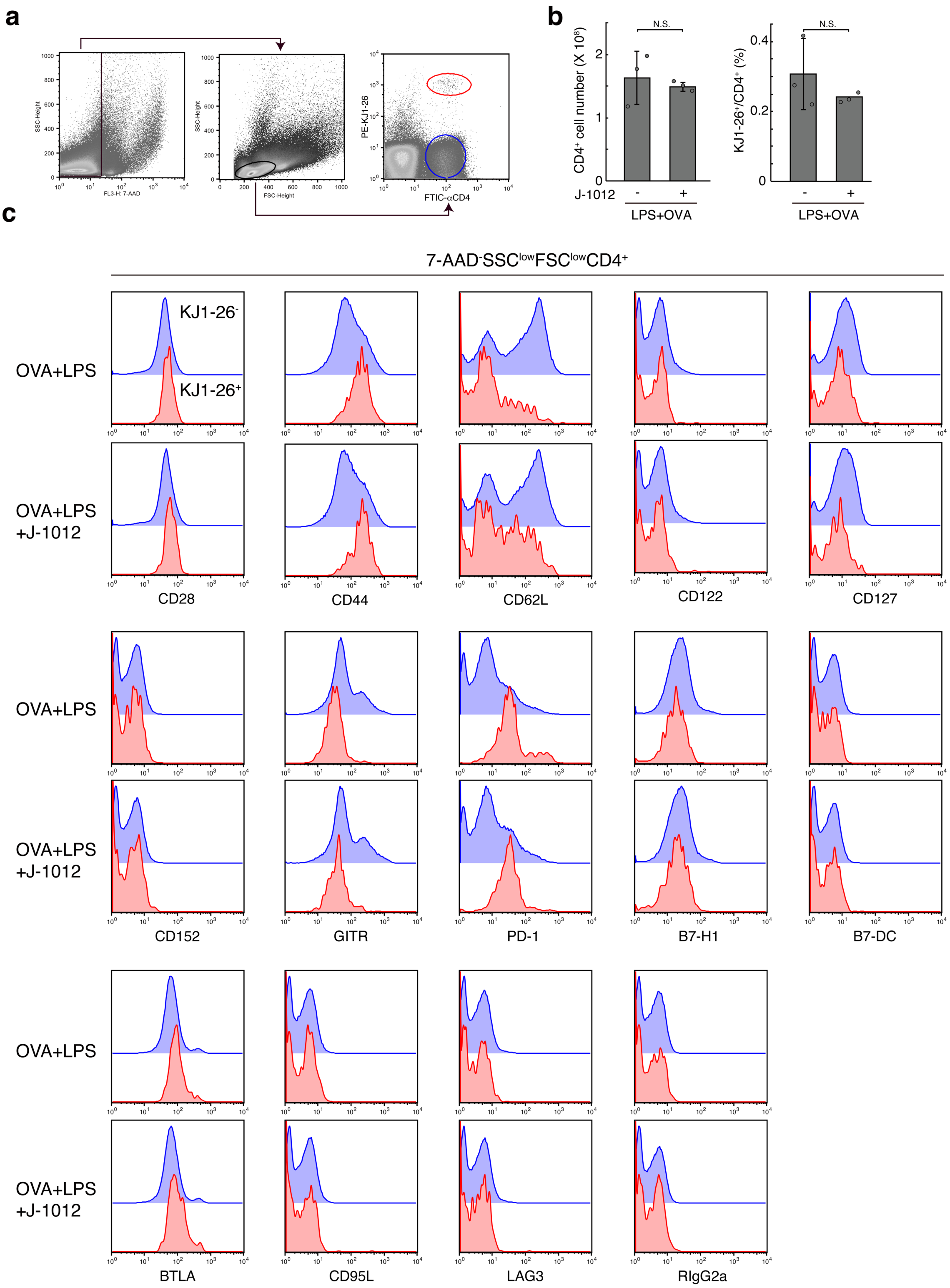

a
b
c
SSC-Height
FL3-H: 7-AAD
FSC-Height
PE-KJ1-26
FTIC-αCD4
CD4+ cell number (X 10^6)
KJ1-26+/CD4+ (%)
N.S.
J-1012
LPS+OVA
7-AAD-SSClowFSClowCD4+
KJ1-26-
KJ1-26+
OVA+LPS
OVA+LPS +J-1012
CD28
CD44
CD62L
CD122
CD127
CD152
GITR
PD-1
B7-H1
B7-DC
BTLA
CD95L
LAG3
RIgG2a

Supplementary Figure 10| Effects of J-1012 N-glycan on transferred DO11.10 T cells after septic inflammation.

Analyses of transferred DO11.10 T cells after 2 weeks of sepsis induction as in Fig. 4d. **a**. Identification of DO11.10 T cells as 7-AAD^{-}CD4^{+}KJ-1-26^{+} cells. **b.** CD4^{+} T cells (*left panel*) and DO11.10 CD4 T cells (*right panel*) in spleen. Data are expressed as the mean ± SD (n=3). **c.** Expression of various types of surface markers on CD4^{+}KJ1-26^{-} (*blue*) and CD4^{+}KJ1-26^{+} (*red*) T cells. Data are representative of two independent experiments. N.S., not significant by unpaired two-tailed Student's *t*-test. ($p > 0.5$)

a

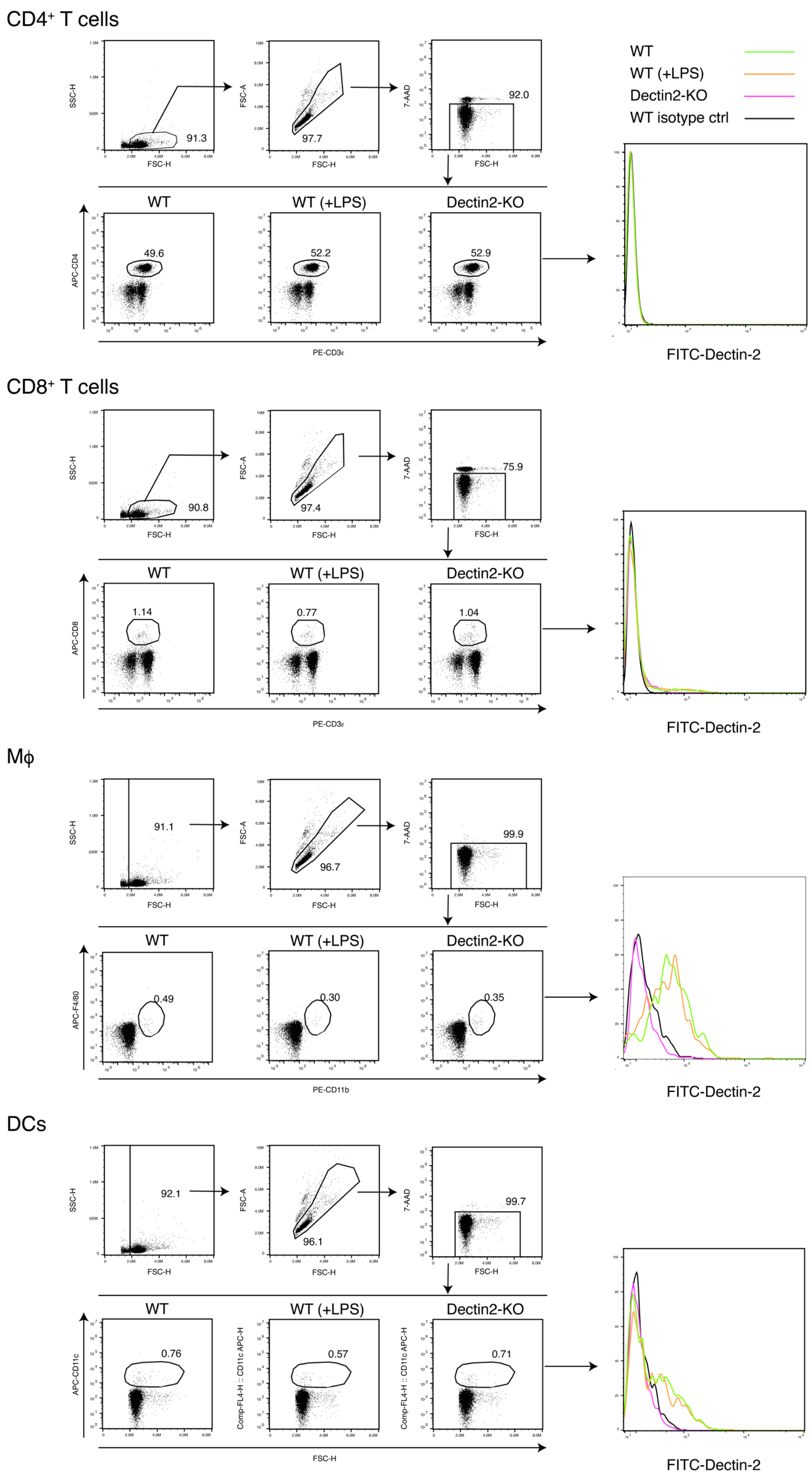
CD4+ T cells
91.3
97.7
92.0
WT
WT (+LPS)
Dectin2-KO
49.6
52.2
52.9
APC-CD4
PE-CD3ε
FITC-Dectin-2
WT isotype ctrl
CD8+ T cells
90.8
97.4
75.9
1.14
0.77
1.04
APC-CD8
Mϕ
91.1
96.7
99.9
0.49
0.30
0.35
APC-F4/80
PE-CD11b
DCs
92.1
96.1
99.7
0.76
0.57
0.71
APC-CD11c
Comp-FL4-H :: CD11c APC-H
SSC-H
FSC-A
FSC-H
7-AAD

b

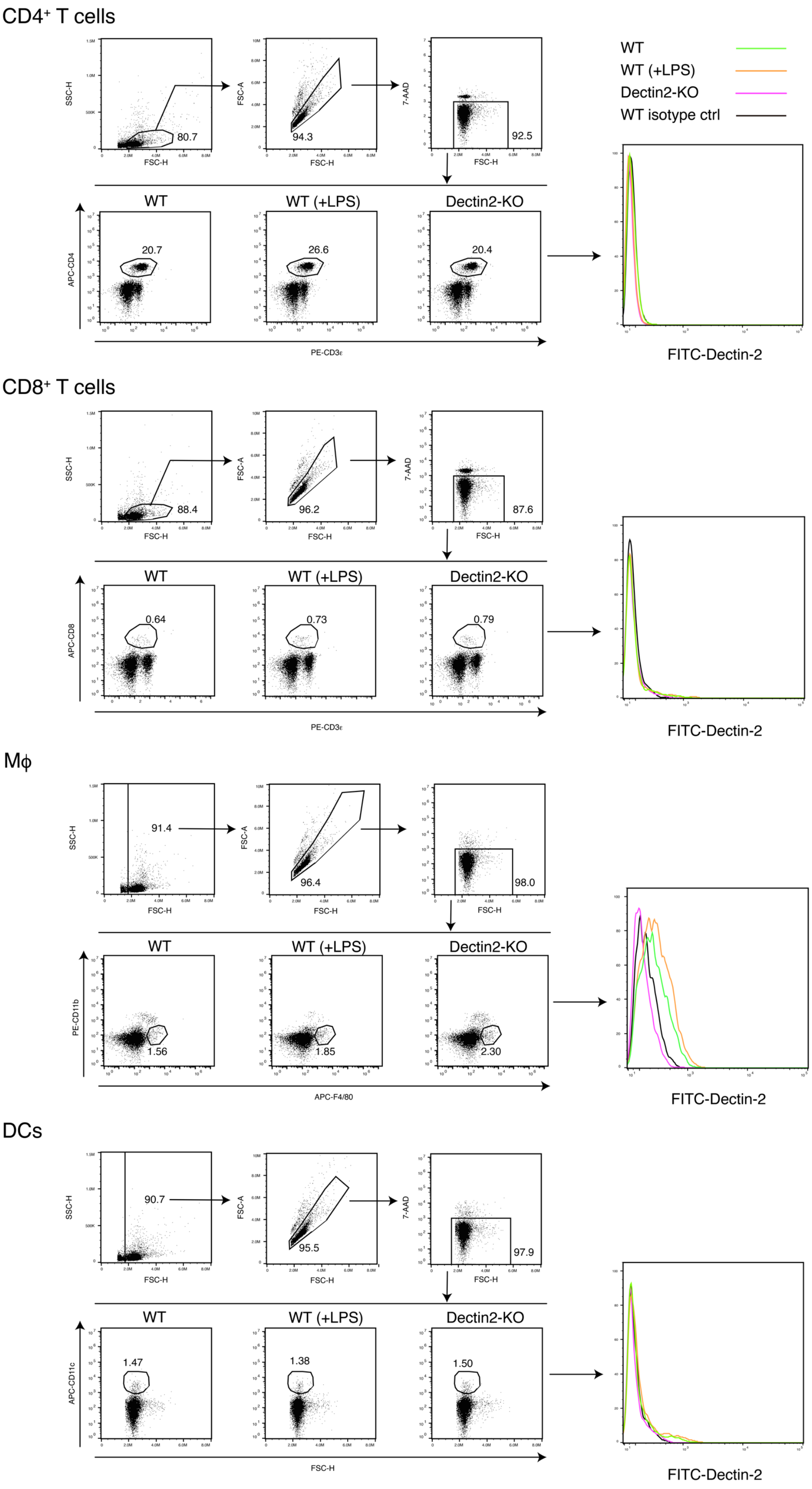
CD4⁺ T cells
CD8⁺ T cells
Mϕ
DCs
WT
WT (+LPS)
Dectin2-KO
WT isotype ctrl
SSC-H
FSC-H
FSC-A
7-AAD
APC-CD4
APC-CD8
PE-CD3ε
PE-CD11b
APC-F4/80
APC-CD11c
FITC-Dectin-2
80.7
94.3
92.5
20.7
26.6
20.4
88.4
96.2
87.6
0.64
0.73
0.79
91.4
96.4
98.0
1.56
1.85
2.30
90.7
95.5
97.9
1.47
1.38
1.50

Supplementary Figure 11| Expression of Dectin-2 in Mϕ, DCs and T cells. Single cells from superficial lymph nodes **(a)** and spleen **(b)** of WT and Dectin-2KO mice were analyzed by flow cytometry. Mϕ, DCs and T cells were identified as 7-AAD^{-}CD11b^{+}F4/80^{+}, 7-AAD^{-}CD11c^{+} and 7-AAD^{-}CD3ε^{+}CD4^{+}/CD8^{+}, respectively. Cells from WT mice pre-treated with *i.v.* injection of LPS (15 μg/20 g body weight) for 2 h were also analyzed. Data are representative of two independent experiments.

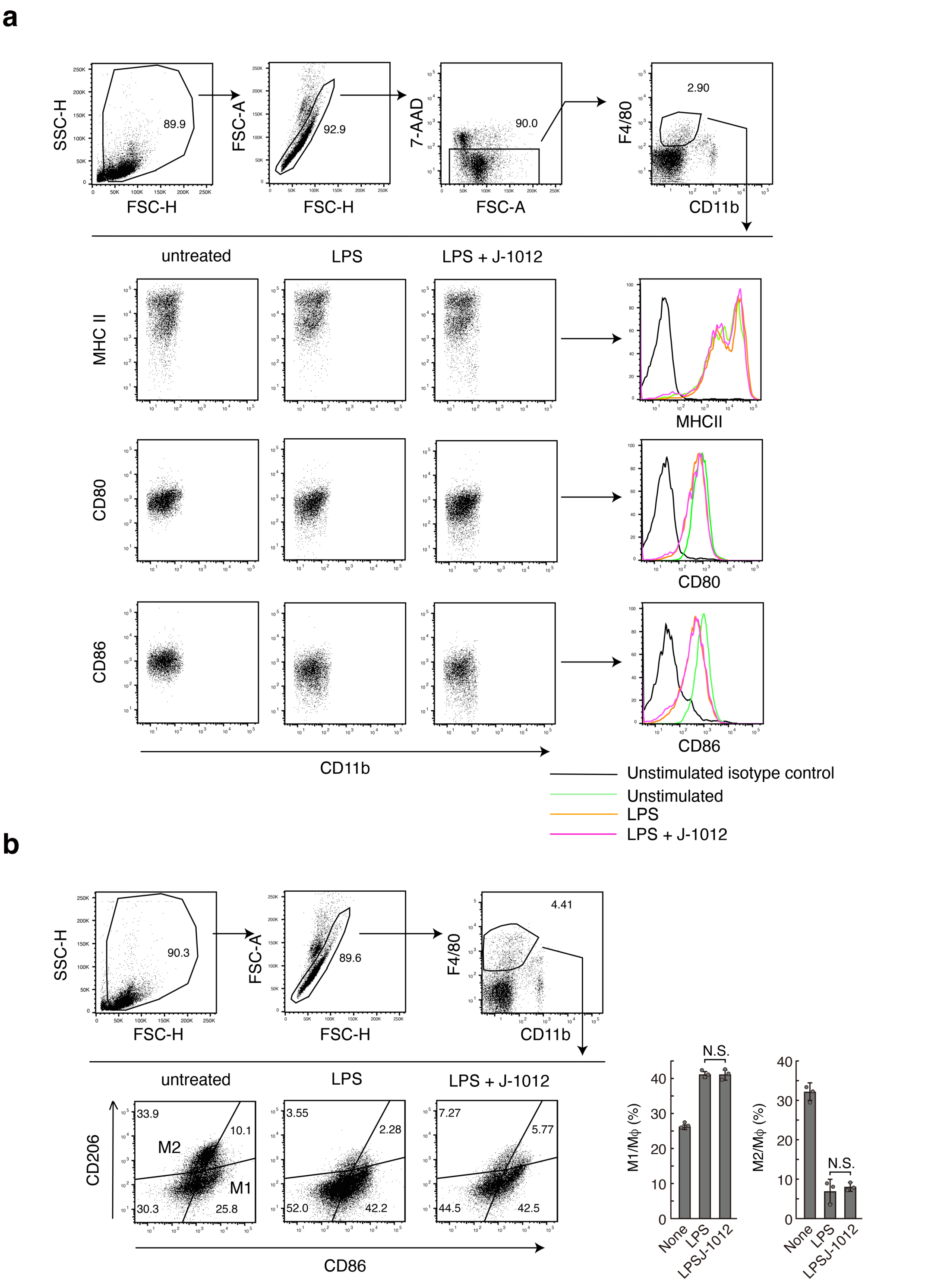

Supplementary Figure 12| Properties of splenic Mϕ after treatment with LPS and J-1012 N-glycan. (**a**) Mice were treated as in Fig. 4b without OVA. After 2 weeks, expression of MHC class II, CD80 and CD86 of Mϕ (AAD$^-$CD11b$^+$F4/80$^+$) in spleen was analyzed by flow cytometry. (**b**) The ratio of M1 and M2 type Mϕ was analyzed in accordance with expression of CD206 and CD86. Data are expressed as the mean ± SD

(n=3) and are representative of at least two independent experiments. N.S., not significant by unpaired two-tailed Student's *t*-test. ($p > 0.5$)

Supplementary Table I. Inhibitors used in this study.

Inhibitor	Target	Concentration	supplier	number
Genistein	Tyrosine kinase	10 μM	Sigma-Aldrich	G6649
Herbimycin A	Tyrosine kinase	25 μM	Sigma-Aldrich	H6649
PP2	Src kinase	10 μM	Sigma-Aldrich	P0042
PP3	PP2 control	10 μM	Calbiochem	529574
DAG kinase inhibitor I	DAG kinase I	10 μM	Sigma-Aldrich	D5919
Rottlerin	PKC	10 μM	Calbiochem	557370
AKT inhibitor	AKT1/2	5 μM	Sigma-Aldrich	A6730
SB 203580	p38	20 μM	Calbiochem	559389
PDTC	NF-kB	50 μM	Sigma-Aldrich	P-8765
SP600125	JNK	10 μM	Sigma-Aldrich	S5567
Wortmannin	PI3K	0.5 μM	Sigma-Aldrich	W1628
L-744.832	CaMK	10 μM	Calbiochem	42270
NF-AT activation inhibitor III	NF-AT	17.6 μM	Calbiochem	480403
GW5074	Raf-1	1 μM	Sigma-Aldrich	G6416
Piceatannol	Syk	25 μM	Sigma-Aldrich	P0453
