# Supplementary material for: Cell wall N-glycan of Candida albicans ameliorates early hyper- and late hypo-immunoreactivity in sepsis
Source: Commun Biol. 2021 Mar 16;4:342. doi: 10.1038/s42003-021-01870-3 (PMC7966402; doi:10.1038/s42003-021-01870-3)
Supplement: Supplementary file 2 — Description of Additional Supplementary Files [file 42003_2021_1870_MOESM2_ESM.pdf]

Description of Additional Supplementary Files

File Name: Supplementary Data 1

Description: Source data for Fig. 1

File Name: Supplementary Data 2

Description: Source data for Fig. 2

File Name: Supplementary Data 3

Description: Source data for Fig. 3

File Name: Supplementary Data 4

Description: Source data for Fig. 4

File Name: Supplementary Data 5

Description: Source data for Supplementary Fig. 1, 2, 5, 7, 8, 9, 10 and 12
