# Supplementary material for: Cell wall N-glycan of Candida albicans ameliorates early hyper- and late hypo-immunoreactivity in sepsis
Source: Commun Biol. 2021 Mar 16;4:342. doi: 10.1038/s42003-021-01870-3 (PMC7966402; doi:10.1038/s42003-021-01870-3)
Supplement: Supplementary file 8 — Reporting Summary [file 42003_2021_1870_MOESM8_ESM.pdf]

nature research

Corresponding author(s): Kazuhiko Takahara

Last updated by author(s): 2021/1/28

Reporting Summary

Nature Research wishes to improve the reproducibility of the work that we publish. This form provides structure for consistency and transparency in reporting. For further information on Nature Research policies, see our Editorial Policies and the Editorial Policy Checklist.

Statistics

For all statistical analyses, confirm that the following items are present in the figure legend, table legend, main text, or Methods section.

n/a	Confirmed	
☐	☒	The exact sample size (*n*) for each experimental group/condition, given as a discrete number and unit of measurement
☐	☒	A statement on whether measurements were taken from distinct samples or whether the same sample was measured repeatedly
☐	☒	The statistical test(s) used AND whether they are one- or two-sided<br>*Only common tests should be described solely by name; describe more complex techniques in the Methods section.*
☒	☐	A description of all covariates tested
☒	☐	A description of any assumptions or corrections, such as tests of normality and adjustment for multiple comparisons
☐	☒	A full description of the statistical parameters including central tendency (e.g. means) or other basic estimates (e.g. regression coefficient) AND variation (e.g. standard deviation) or associated estimates of uncertainty (e.g. confidence intervals)
☒	☐	For null hypothesis testing, the test statistic (e.g. *F*, *t*, *r*) with confidence intervals, effect sizes, degrees of freedom and *P* value noted<br>*Give P values as exact values whenever suitable.*
☒	☐	For Bayesian analysis, information on the choice of priors and Markov chain Monte Carlo settings
☒	☐	For hierarchical and complex designs, identification of the appropriate level for tests and full reporting of outcomes
☒	☐	Estimates of effect sizes (e.g. Cohen's *d*, Pearson's *r*), indicating how they were calculated

Our web collection on statistics for biologists contains articles on many of the points above.

Software and code

Policy information about availability of computer code

Data collection	1. CellQuest http://www.bdbiosciences.com/
Data analysis	1. FlowJo; V8-V10.6. https://www.flowjo.com<br>2. Microsoft Excel v16 https://www.microsoft.com

For manuscripts utilizing custom algorithms or software that are central to the research but not yet described in published literature, software must be made available to editors and reviewers. We strongly encourage code deposition in a community repository (e.g. GitHub). See the Nature Research guidelines for submitting code & software for further information.

Data

Policy information about availability of data

All manuscripts must include a data availability statement. This statement should provide the following information, where applicable:

- Accession codes, unique identifiers, or web links for publicly available datasets
- A list of figures that have associated raw data
- A description of any restrictions on data availability

All data are available in the manuscript.

Field-specific reporting

Life sciences study design

All studies must disclose on these points even when the disclosure is negative.

Sample size	No statistical methods were used to determine sample size.
Data exclusions	No data were excluded from the analyses.
Replication	All experiments were performed at least 2 times and showed consistent results, as reported i the manuscript.
Randomization	This study did not contain experiment that required randomized.
Blinding	Blinding was not relevant in this study, because all the data were analyzed using unbiased methods.

Reporting for specific materials, systems and methods

We require information from authors about some types of materials, experimental systems and methods used in many studies. Here, indicate whether each material, system or method listed is relevant to your study. If you are not sure if a list item applies to your research, read the appropriate section before selecting a response.

Materials & experimental systems

n/a	Involved in the study	
☐	☒	Antibodies
☒	☐	Eukaryotic cell lines
☒	☐	Palaeontology and archaeology
☐	☒	Animals and other organisms
☒	☐	Human research participants
☒	☐	Clinical data
☒	☐	Dual use research of concern

Methods

n/a	Involved in the study	
☒	☐	ChIP-seq
☐	☒	Flow cytometry
☒	☐	MRI-based neuroimaging

Antibodies

Antibodies used

1) From eBioscience (San Diego, CA),
Biotin-labeled anti-B7-H1 (MIH15), 13-5982-85
Biotin-labeled anti-B7-DC (TY25), 13-5986
Biotin-labeled anti-BTLA (6F7), 13-5950
Biotin-labeled anti-CD8 (53-6.7), 13-0081
Biotin-labeled anti-CD44 (1M7), 13-0441-85
Biotin-labeled anti-CD95L (MFL3), 13-5911
Biotin-labeled anti-CD152 (UC10-4B9), 13-1522
Biotin-labeled anti-GITR (DTA-1), 13-5874
Biotin-labeled anti-PD-1 (J43) , 13-9985
Biotin-labeled ratIgG2aκ (eBR2a),13-4321-85
Biotin-labeled Armenian hamster IgG (eBio299Arm),13-4888-81
FITC-labeled anti-CD4 (GK1.5), 11-0041
FITC-labeled F4/80 (BM8), 11-4801-85
APC-labeled anti-CD11c (N418), 17-0114-82
APC-labeled anti-MHC class ∘ (M5/114.15.2), 17-5321-81
PE-labeled antiCD3e (145-2C11), 12-0031-85
PE-labeled anti-CD80 (16-10A1),12-0801-83
PE-labeled ratIgG2aκ(eBR2a, 12-4321-83

2) From Miltenyi Biotec (Bergisch Gladbach, Germany)
FITC-anti-mouseDectin-2 (KVa7-6E7),130-102-212

3) From BD Pharmingen (La Jolla, CA).
Biotin-labeled anti-CD4 (GK1.5), 09422D
Biotin-labeled anti-CD11c (N418), 553801
Biotin-labeled anti-CD16/32 (2.4G2), 01242D
Biotin-labeled anti-CD19 (1D3), 09652D
Biotin-labeled anti-CD28 (37.51), 553296
Biotin-labeled anti- CD49b (DX5), 553856
Biotin-labeled anti- CD80 (16-10A1), 553767

Biotin-labeled anti- CD86 (GL1), 553690
Biotin-labeled anti-CD127 (B12-1), 555288
Biotin-labeled anti-B220 (RA3-6B2), 01122D
Biotin-labeled anti-Gr-1 (RB6-8C5), 553124
Biotin-labeled anti-I-Ad (AMS-32.1), 553546
Biotin-labeled anti-IL-10 (JES5-16E3), 18432D
Biotin-labeled anti-LAG3 (C9B7W), 123206
Biotin-labeled anti-Mac1 (M1/70), 553309
Biotin-labeled anti-gdTCR (GL3), 01312D
FITC-labeled control RatIgG2a (R35-95)
PE-labeled anti-DO11.10 TCR (KJ1-26), 551772
PE-labeled anti-CD11b (M1/70), 55739
PE-labeled anti-CD86 (GL1), 553692
PE-labeled hamsterIgG1λ1 (G235-2356), 553954
APC-labeled anti-CD4 (RM4-5), 553051

4) BioLegend (San Diego, CA)
FITC-labeled anti-CD11c (N418), 117306
Alexa488-labeled anti-CD206 (C068C2), 141710
APC-labeled F4/80 (BM8), 123116
APC-labeled ratIgG2aκ (RTK2758), 400512
PE-Cy7-labeled streptavidin, 405206

5) TOMBO biosciences
APC-labeled anti-CD8a (53-6.7), 20-0081-U100

6) MBL (nagoya, Japan)
Biotin-anti-Mincle (1B6), D266-6

7) The Jackson Laboratory
Alexa647-labeled streptavidin, AB_2341101

Validation	All antibodies were well-established clone and commercially available from companies described above. All antibodies were used at concentrations recommended by the companies. The validation data for the antibodies and product information are provided on the companies' website.

Animals and other organisms

Policy information about studies involving animals; ARRIVE guidelines recommended for reporting animal research

Laboratory animals	Both female and male Balb/c background wild type, Dectin-1KO and Dectin-2KO mouse were used.
Wild animals	No wild animal was used.
Field-collected samples	No Field-collected sample was used.
Ethics oversight	All experiments were conducted according to our institutional guidelines on Kyoto University.

Note that full information on the approval of the study protocol must also be provided in the manuscript.

Flow Cytometry

Plots

Confirm that:

☐ The axis labels state the marker and fluorochrome used (e.g. CD4-FITC).

☒ The axis scales are clearly visible. Include numbers along axes only for bottom left plot of group (a 'group' is an analysis of identical markers).

☒ All plots are contour plots with outliers or pseudocolor plots.

☐ A numerical value for number of cells or percentage (with statistics) is provided.

Methodology

Sample preparation	For flow cytometry, single cells from lymph node were obtained using metal mesh and syringe plunger.
Instrument	For flow cytometry, cells were acquired with an FACSCalibur and Accuri C6 Plus (BD Biosciences) cell analyzer.

Software	1. CellQuest http://www.bdbiosciences.com/<br>2. FlowJo; V8-V10.6. https://www.flowjo.com
Cell population abundance	1) Macrophage purified from resident peritoneal cells by magnetic sorting: more than 95% as CD11b-positive cells<br>2) Bone marrow-derived DCs purified from bone marrow culture with GM-CSFby magnetic sorting: more than 95% as CD11c-positive cells<br>3) DO11.10 cells purified from DO11.10 transgenic mouse lymph nodes by magnetic sorting: more than 85% as KJ1-26-positive cells
Gating strategy	All gating strategy was described in the supplemental figure.

☒ Tick this box to confirm that a figure exemplifying the gating strategy is provided in the Supplementary Information.
